# Quarantine and its legal dimension

**DOI:** 10.3906/sag-2004-153

**Published:** 2020-04-21

**Authors:** Rahmi KILIÇ, Çiğdem ATAMAN HATİPOĞLU, Cemil GÜNEŞ

**Affiliations:** 1 Department of Ear Nose Throat Clinic, Ankara Training and Research Hospital,Health Sciences University, Ankara Turkey; 2 Department of Infectious Diseases and Clinical Microbiology Clinic,Ankara Training and Research Hospital,Health Sciences University, Ankara Turkey; 3 Health Ministry, General Directorate of Legal Services, Ankara Turkey

**Keywords:** Quarantine, isolation, law

## Abstract

Quarantine and isolation are public health measures used for centuries to prevent the spread of infectious diseases. Quarantine is separation of persons who have been exposed to an infection but asymptomatic, while isolation is separation of infected patients. Voluntary quarantine is preferred, but if necessary, it can be mandatory. These implementations can lead to restrictions on individual liberties, leading to ethical and legal problems. Isolation and quarantine enforcement are regulated by laws. Those who do not follow the quarantine rules could be punished. Isolation and quarantine practices in our country are described in General Hygiene Law. In this study the importance of quarantine, when and how it is applied, and its ethical and especially legal dimension are discussed.

With the COVID 19 pandemic, which started in China at the end of 2019 and affected the whole world, the measures to be taken to control the epidemics became a current issue. Quarantine is one of the most effective public health measures for controlling outbreaks. It means “separation and restriction of the movement of people who are exposed to a contagious disease” as stated by Centers for Diseases Control and Prevention (CDC) [1].The aim of the quarantine is to monitor the exposed person for the development of symptoms and to prevent the possible transmission of the pathogen from the asymptomatic person to others. Quarantine may be voluntary or mandatory, voluntary quarantine is preferred. During the quarantine, the individual must remain in his home or in a place designated for this task and follow the rules. It may be applied to an individual or a community and must continue until the longest incubation period of the pathogen [2]. In some articles, “precautionary self-isolation of contacts” is used instead of quarantine [3]. Isolation means “separation of sick people with a contagious disease from people who are not sick”. The aim of this measure is to prevent or minimize person-to-person transmission of disease. Although the quarantine and isolation definitions are different, these two terms can often be used one for the other. Briefly, while quarantine is applied to asymptomatic patients, isolation is applied to symptomatic patients. If the person becomes symptomatic during the quarantine period, isolation should be started. [1,4,5].

Quarantine of the asymptomatic immigrants who came from countries where epidemics occur is an effective preventive method and has been applied for many years [6]. The CDC routinely checks passengers arriving at land border crossings and ports of entry for contagious diseases [1].

It is known that the quarantine was first applied during the bubonic plague epidemic, also called black death, in the 14th century. In order to prevent the coastal cities from being affected by the plague epidemic, ships arriving in Venice were kept in the harbor for 40 days, then they could approach the shore. The term quarantine was derived from Italian word quaranta that means forty [1,7,8]. Then islands in Italy were used quarantine stations for controlling the plague and leprosy during the epidemics [9]. After the yellow fever and cholera epidemics in the late 1800s, quarantine laws were passed for the first time in America [10]. Quarantine is also an important component to fight influenza outbreaks. During the Spanish flu pandemic in 1918–1919, isolation and quarantine measures were widely used to prevent transmission [1].

During the SARS outbreak in 2003, quarantine was applied successfully along with other control measures. Level A quarantine measures which implemented for persons who have contact with suspected SARS patients and Level B quarantine measures for travelers came from SARS affected regions were effective to control the outbreak in Taiwan [11]. In a retrospective modeling study, applying the level A and level B quarantine rules together during the SARS outbreak has been shown to reduce the number of cases and mortality by half. It stated in this study that this combined application may be useful especially in outbreaks that will develop with new or not well-known infectious diseases [12].

Isolation and quarantine procedures were widely used too in Ebola outbreak in 2014–2015 for preventing humanto-human transmission [13]. Due to the risk of close contact with infected patients during their work, quarantine may also be required for healthcare professionals. In fact, health care workers returning from the regions affected by Ebola were asked to remain in quarantine for 21 days by some governors. But it was not supported scientifically, because asymptomatic persons were not contagious [14].

Health measures including isolation, quarantine, social distance, and community containment play an important role in the fight of current COVID-19 outbreak. Along with other control measures, China implemented the largest quarantine in history and managed to control the outbreak [15].

Although the measures taken to control the outbreaks are in the public interest, they may be the subject of debate because of the potential of restriction of individual liberties. For this reason, applications such as quarantine and isolation should be examined in terms of political, ethical, legal, and socioeconomic aspects besides public health [16,17].

When applying quarantine, it is necessary to pay attention to some ethical rules. Necessary precautions should be taken to avoid discrimination and stigmatization during the quarantine and isolation applications, as previously experienced in the past, in patients with bubonic plague, syphilis, gonorrhea and HIV infection [18–20]. Quarantine and isolation being the practices that limit liberty of the individuals must have some criteria for ethical acceptance. Firstly, other people should be harmed when this restriction was not applied. Least restrictive measures should be taken to control the spread of the disease and these measures should be voluntary. Medical and social needs of the quarantined person must be met, and the application of restriction should be equally fair and transparent for all people [17,21].

When deciding on the quarantine application, it is necessary to think that it can cause negative results. If the rules are not followed during the application especially during the restrictions imposed on many people, such as quarantine practice in the hospital, some risks may arise. One of these risks is the possibility of transmissionof the agent. If someone who develops symptoms during quarantine is not isolated, it can infect another person in quarantine. The same risk exists in quarantine applications for diseases that are at risk of transmission begin before symptoms. Moreover, difficulties may arise during the cohort of those with and without signs of infection. Patients admitted to the hospital with other diseases and conditions, is another problem. Apart from the risk of transmission of the contagious agent to these patients, it is an important issue that they do not receive adequate service regarding their actual diseases. In addition, all medical and human needs of healthcare professionals and patients must be met during the hospital quarantine [4]. Another important risk in the quarantine process is psychological problems. Both healthcare professionals and patients are afraid of becoming infected, but also worry about infecting their families and friends [4–22]. 

Although its important role in limiting outbreaks, quarantine implementation has some difficulties (Figure). Quarantined persons will need psychological support, food and water, and household and medical supplies. Financial compensation for workdays lost should be considered, law enforcement may need to be considered if quarantine violations occur frequently [15].

**Figure 1:**
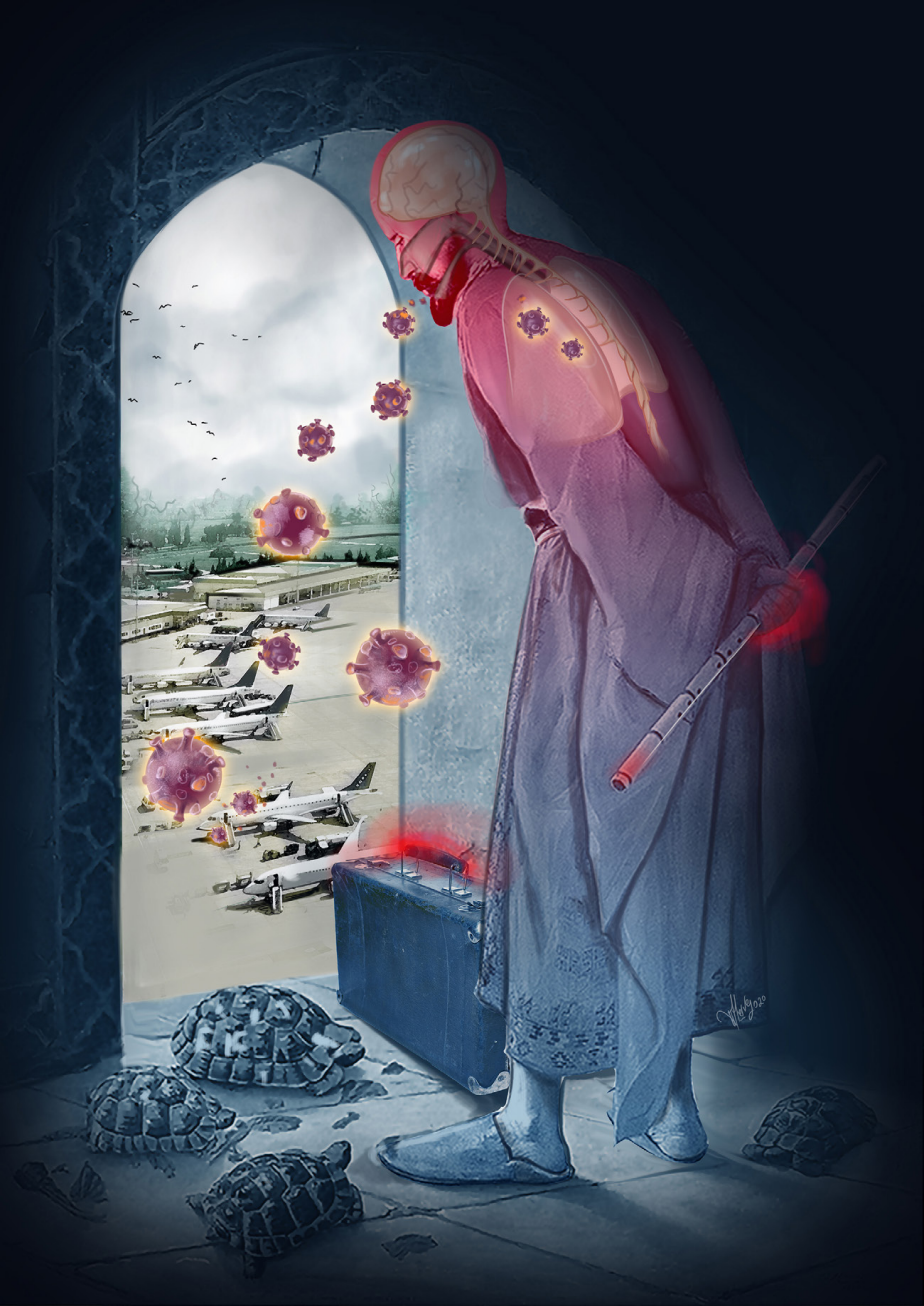
Quarantine is not always easy but should be implemented if necessary (Courtesy of Merve Evren, Visuluma Sceintific Visualisation).

In the study evaluating how the use the quarantine measures, some conditions for a successfully quarantine application was stated. These conditions include the knowledge of people or community that their safety is in danger because of this disease, the trust of people that these efforts will diminish the transmission of the disease and the readiness of people or community to make sacrifices. They state also that government must use effective and proven quarantine methods [23].

The legal dimension of quarantine and isolation practices is also important. According to the federal law in United States (US), quarantine and isolation can be applied for some infectious diseases including cholera, plague, smallpox, diphtheria, yellow fever, viral hemorrhagic fevers, severe acute respiratory syndrome, and pandemic flu for the benefit of society. Responsibility for the efforts to prevent infectious diseases to entering and spreading into the country is given to the CDC. If necessary, the CDC may decide to isolation or quarantine. There are federal and local laws regulating isolation and quarantine enforcement. Sometimes police forces may be needed to enforce these rules. Those who do not follow the quarantine rules can be punished. Individuals can be released from quarantine with the permit of Federal laws [1]. In a study evaluating quarantine and isolation laws in 50 states of US; it stated that only 10 states have minor changes in their isolation and quarantine rules after 2014–2016 Ebola outbreaks. In remaining states, quarantine rules have not been changed for a long time. It was observed in this study that there were considerable differences between the states in terms of quarantine and isolation rules. In 51% of states, the use of police forces was permitted, if necessary, to protect the public’s health. In 45% of the states, financial support was planned and provided for the safe and humane quarantine process. Only 20% had protective rules to prevent the individual in quarantine from losing their job [24].

In our country, the General Hygiene Law (Umumi Hıfzıssıhha Kanunu) No. 1593 is accepted as the constitution of healthcare services. In the various articles of the General Hygiene Law, quarantine application was included within the scope of “combating contagious and epidemic diseases” and the word isolation was used instead of the word quarantine [25].

There are regulations regarding the isolation practices in the scope of fight against epidemics in the before mentioned Law. Isolation practices for people living in our country are described in Article 72 of this Law, isolation practices for passengers coming to the country by ship in Article 49, by land border gates in Article 54 and by air in Article 56. In addition, in the 72nd Article of the same Law, there is a provision regarding the quarantine of a certain region or the evacuation of people from this region in case of a possible or confirmed epidemic disease [25].

Authority to decide on quarantine implementation and other measures within the scope of combating epidemic diseases is given to the Ministry of Health for the country in general by the Article 64 of the Law numbered 1593. This authority for the local level measures is given to the provincial and district public sanitation boards by the Article 27 of the same Law [25].

In the event of a seriously dangerous infectious disease, the guardianship court stated that freedom of liberty could be restricted for the treatment of individuals within the scope of Article 432 of the Turkish Civil Code No. 4721, but there is no doubt that the provisions of the Law No. 1593, which is a special law, should be applied in cases of outbreak [25,26].

Within the scope of combating epidemic diseases, strict rules-related measures such as quarantine can be applied, or it is possible to take measures that are not as strict as quarantine in the form of home observation, and that people are allowed to leave home in a controlled situation. Sanctions to be applied in case of failure to comply with these measures vary according to the type of measure. In accordance with the principle of legal security, it is necessary to provide written documents containing these issues to the relevant people in return for signature in order to ensure that they know the type of measure applied against them and the sanction to be applied in case of violation of the measure. It is important to provide signed documents for future legal disputes in terms of proof of law.

In case of failure to comply with the quarantine decision, the penalties specified in the law are applied about the person concerned. According to the Article 284 of the General Hygiene Law No. 1593, persons who oppose officials authorized to conduct investigations on infectious diseases are punished in accordance with Article 195 of the Turkish Criminal Code. According to the Article 195 of the Turkish Criminal Code No. 5237 titled “Behaving contrary to the measures related to infectious diseases”, the person who does not comply with the measures taken by the competent authorities to quarantine the location of anyone who has contracted or died of an infectious disease, is punished with imprisonment from two months to a year [25,27].

In case of acting contrary to the observation measure at home, a fine is applied to the person concerned. In accordance with Article 282 of the General Hygiene Law No. 1593, those who act against the prohibitions written in the Law or who do not comply with the obligations shall be fined from 250 Turkish Liras (TL) to 1000 TL if their acts do not constitute a crime. When revaluation rates are applied, the current amount of the mentioned penalty for 2020 is 789–1380 TL ($115–202). In case people do not comply with the observation measure at home, it is possible to be punished with administrative fines, as well as to be placed in quarantined areas [25].

As a result, quarantine, which is an old practice, is still applied today with other control measures to prevent the spread of communicable diseases. We think that each healthcare worker should know the legal and ethical aspects of quarantine and isolation practices as well as public health effects.

## Acknowledgments/disclaimers

Prof. Dr. Rahmi KILIÇ is a member of COVID-19 Advisory Committee of Ministry of Health of Turkey. Prof. Dr. Çiğdem ATAMAN HATİPOĞLU is working in the pandemic hospital that was used to quarantine the Turkish Citizens evacuated from Wuhan.
